# Heavy Metals and Human Health: Mechanistic Insight into Toxicity and Counter Defense System of Antioxidants

**DOI:** 10.3390/ijms161226183

**Published:** 2015-12-10

**Authors:** Arif Tasleem Jan, Mudsser Azam, Kehkashan Siddiqui, Arif Ali, Inho Choi, Qazi Mohd. Rizwanul Haq

**Affiliations:** 1 School of Biotechnology, Yeungnam University, Gyeongsan 712-749, Korea; 2 Department of Biosciences, Jamia Millia Islamia, New Delhi 110025, India; azammudsser@gmail.com (M.A.); kehkashansiddiqui7@gmail.com (K.S.); ali.arifali@gmail.com (A.A.); haqqmr@gmail.com (Q.M.R.H.)

**Keywords:** biomolecules, dietary antioxidants, free radicals, heavy metals

## Abstract

Heavy metals, which have widespread environmental distribution and originate from natural and anthropogenic sources, are common environmental pollutants. In recent decades, their contamination has increased dramatically because of continuous discharge in sewage and untreated industrial effluents. Because they are non-degradable, they persist in the environment; accordingly, they have received a great deal of attention owing to their potential health and environmental risks. Although the toxic effects of metals depend on the forms and routes of exposure, interruptions of intracellular homeostasis include damage to lipids, proteins, enzymes and DNA via the production of free radicals. Following exposure to heavy metals, their metabolism and subsequent excretion from the body depends on the presence of antioxidants (glutathione, α-tocopherol, ascorbate, *etc.*) associated with the quenching of free radicals by suspending the activity of enzymes (catalase, peroxidase, and superoxide dismutase). Therefore, this review was written to provide a deep understanding of the mechanisms involved in eliciting their toxicity in order to highlight the necessity for development of strategies to decrease exposure to these metals, as well as to identify substances that contribute significantly to overcome their hazardous effects within the body of living organisms.

## 1. Introduction

Technological advancements that led to improved life standards, has raised new challenges with respect to environmental safety, as unrestrained industrialization and urbanization without proper emission controls and pollution abatement have put human lives at risk [1]. In developing countries, a need for economic growth that generally relies on agricultural and industrial development has bypassed environmental protection guidelines to a greater extent [2,3]. Heavy metals including metalloids, such as arsenic, are widely utilized to sustain the living standards of the modern world. In addition to natural sources, the amount of metals entering the environment through anthropogenic activities ranging from coal driven power plants to waste incinerators has increased tremendously. Owing to their prevalence, risk of human exposure to such metals continues to increase. In the past, inadequate regulation of recycling programs has led to accidental exposure. For example, the Itai-Itai and Minimiata disasters threatened public health and raised concerns regarding their safety in the environment. Because water and soil pollution represent the most important problems being faced by both developed and developing countries, metals in the environment are now widespread environmental contaminants that pose a continuous threat to mankind. Overall, metal toxicity arising from different sources is a problem of increasing significance from an ecological, evolutionary, nutritional, and environmental perspective [4,5].

Heavy metal contamination is increasingly being recognized as dramatic in large parts of the developing world, particularly in India and China [6,7]. Contamination of dietary substances by chemicals and non-essential elements such as heavy metals is known to have a series of adverse effects on the body of humans and animals [8]. Because they are ubiquitous and recalcitrant, their entry into the body poses a potential health risk to human populations. Metals can escape control mechanisms such as homeostasis, transport, compartmentalization, and binding to specified cell constituents, thus they can have toxic and even lethal effects. Heavy metals can cause malfunctioning of the cellular processes via displacement of essential metals from their respective sites. Oxidative deterioration of biological macromolecules has been found to be primarily due to binding of metals to DNA and nuclear proteins [9]. Symptoms are often the first indicators of contamination, and as such help to identify the contaminant. Symptoms that arise as a result of metal poisoning include intellectual disability in children, dementia in adults, central nervous system disorders, kidney diseases, liver diseases, insomnia, emotional instability, depression and vision disturbances [9,10]. In short, toxicity associated with exposure to metals if unrecognized or inappropriately treated represents a clinically significant medical problem, having greater impact on increasing the morbidity and mortality rate.

Having broader applicability in domestic, industrial, and agricultural purposes, their widespread distribution in the environment raises serious concerns over their potential health effects on humans. Although toxicity that arises from sudden exposure to substantial quantities of metals (such as from occupational exposure) typically affects multiple organ systems, severity of the health outcomes of toxic metals depends on the type and form of the element, route and duration of the exposure, and, to a greater extent, on a person’s individual susceptibility [10]. In terms of health perspective, pathophysiology of metals depends primarily on the generation of oxidative stress, which is characterized by (a) increased Reactive Oxygen Species (ROS) and Reactive Nitrogen Species (RNS) production; (b) depletion of intracellular antioxidant stores and free-radical scavengers; and (c) inhibition or reduction of the activity of enzymes that contribute significantly to the metabolism and detoxification of reactive oxygen species. This review provides comprehensive information regarding the toxicity of different metals and a description of the complexity of redox based homeostasis regulation by various antioxidant systems.

## 2. Heavy Metals

These metallic elements (mercury, arsenic, and lead) that are able to induce toxicity even at lower levels of exposure are considered systemic toxicants. Occupying the top position on the list of hazardous substances, the following sections provide insight into the mechanisms through which these metals exert their toxicity within the body of living organisms.

### 2.1. Mercury (Hg)

Mercury, considered the most toxic heavy metal, has become part of the environment owing to anthropogenic activities including agriculture, municipal wastewater discharge, mining, incineration, and discharges of industrial wastewater [11,12]. Having different bioavailabilities and toxicities associated with them, it exists in nature as an elemental or metallic form, in inorganic salts and as organomercurial compounds. In its elemental (Hg) form, it primarily exists as a liquid metal. Although used extensively in measuring equipment (pyrometers, thermometers, *etc.*), mercury arc and fluorescent lamps, and as a catalyst, its use as a component of batteries, in industries (pulp and paper), and mostly as amalgams in dental preparations is worth mentioning. Metallic mercury finds its entry into (1) air mainly through mining and burning processes; and to (2) water and soil through erosion of natural depots, discharges from industries and runoff from landfill sites. Inside the body, average half-life of inhaled mercury is approximately 60 days [13]. Symptoms attributed to high level exposure to metallic mercury include lung damage (pulmonary toxicity), mucous membrane changes, vomiting, diarrhoea, nausea, skin rashes, increased heart rate or blood pressure (hypertension), renal dysfunction (nephrotoxicity), and severe neurologic abnormalities [14,15]. Intellectual disorder that leads to behavioural changes, anxiety, depression, tremors, and reduced coordination of muscles are common neurological symptoms (Table 1). Inorganic mercury exists either as mercuric (Hg^2+^) or mercurous (Hg^+^) form. Having greater solubility in water, their toxic consequences are much greater compared with the elemental (Hg). Inside the body, it has a half-life of 40 days [16]

Because it is lipophilic in nature, organic mercury can easily permeate across biomembranes. Organic mercury, particularly methylmercury (MeHg), finds its entry through foods such as fish, while ethylmercury can enter the body as part of vaccine preservatives and some antiseptics [17,18]. After its release into the environment (soil and water), inorganic mercury is acted upon by bacteria, leading to its transformation to methylmercury, having the ability to bioaccumulate in fish and other animal tissues [19]. In addition to accumulation of neurotoxic molecules such as aspartate, serotonin, and glutamate, toxicities associated with methylmercury include microtubule destruction, lipid peroxidation and damage to mitochondria. [20]. Compared to methylmercury, ethylmercury is rapidly metabolised into inorganic salts, thereby proceeding through nephrotoxicity [21]. On encountering mercury, humans are reported to develop a disorder, commonly referred to as acrodynia or pink disease [22]. Symptoms associated with this disease include rashes, itching, redness and peeling of the skin from hands, nose and soles of the feet, sleeplessness, and/or weakness. Owing to its health hazardous effects, current levels set by Environmental Protection Act (EPA) and World Health Organization for drinking water are 0.002 mg/L and 0.001 mg/L, respectively [23]. Taken together, increased exposure to mercury, which is known to alter physiological functions in humans, often led to pulmonary toxicity (Elemental form), renal dysfunction (Inorganic) and severe neurologic abnormalities and other intellectual disorders (Organic ones).

#### 2.1.1. Mercury Induced Nephrotoxicity

Though all forms of mercury are toxic, possible health effects vary according to its distribution in the human body. Disparity in its distribution and pattern of biological effect arises due to differences in the transport mechanism and pathway involved in the metabolism [24]. Though dermal contact (~3%) and absorption at the gastrointestinal (GI) tract (<0.1%) contribute to some extent to exposure of elemental mercury, its toxicity is mainly attributed to inhalation (~80%) of elemental mercury vapour at the lung surface [23]. Although brief exposure to high doses results in the toxicity at the lung surface, long term exposure to low doses of elemental mercury have been found to be associated with the toxicity of different organs of the body. Besides facilitating its diffusion across the alveoli surface into circulation, its lipophilic nature makes its passage through the blood–brain barrier into the central nervous system (CNS) and across the placenta. In the circulation, it remains as part of plasma or erythrocytes. On crossing the blood-brain barrier, it gets rapidly converted to inorganic forms, associated with the binding and as such inactivation of enzymes involved in the synaptic and neuromuscular transmission, that often lead to characteristic degenerative changes [22,23]. In erythrocytes, catalase can oxidize elemental mercury to an inorganic metabolite, thereby making it unable to cross the membrane barriers and, as such, follows its toxicity in the form of inorganic mercury.

**Table 1:** Detailed summary of form(s), sources, entry routes, associated symptoms and pronounced health effects corresponding to different metals.

Metal	Form(s)	Sources	Route of Entry	Symptoms		Health Effects	References
				Acute	Chronic		
Mercury, At. No: 80, At. Mass: 200.6	Hg, Hg^2+^, Hg^+^, Hg-organic Oxidation state: +1, +2	Fossil fuel combustion, mining, smelting, solid waste combustion, fertilizers industrial wastewater, use in electrical switches, fluorescent bulbs Mercury arc lamps, incineration of municipal wastes, emissions from mercury products: batteries, thermometers, Mercury amalgams	Inhalation, ingestion and absorption through skin	GI pain, vomiting, diuresis, anemia, hypovolemic shock, renal toxicity, tension, irritability, intention tremors, insomnia, fatigue	Gingivitis, tachycardia, goiter, high urine Hg	Disruption of the nervous system, damage to brain functions, DNA damage and chromosomal damage, allergic reactions, tiredness and headaches, negative reproductive effects, such as sperm damage, birth defects and miscarriages	[22]
Arsenic, At. No: 33, At. Mass: 74.92	As^III^, As^V^, Oxidation state: +3, +5	Pesticides, mining, smelting of gold, Lead, Copper and Nickel, Production of iron and steel, combustion of coal, tobacco smoke	Inhalation and ingestion	Mucosal damage, hypovolemic shock, fever, sloughing, gastro-intestinal pain, anorexia	Weakness, hepatomegaly, melanosis, arrhythmias, peripheral neuropathy, peripheral vascular disease, carcinogenicity, liver angiosarcoma, skin and lung cancer	Birth defects, Carcinogen: lung, skin, liver, bladder, Kidneys, Gastrointestinal damage, Severe vomiting, diarrhea, death	[25]
Lead, At. No: 82, At. Mass: 207.19	Pb^2+^, Oxidation state: +2, +4	Application of lead in gasoline, fuel combustion, industrial processes, solid waste combustion, used in paints, used in ceramics and dishware, Lead is used in some types of PVC mini-blinds	Inhalation and ingestion	Nausea, vomiting, thirst, diarrhea/constipation, abdominal pain, hemoglobinuria, oligura leading to hypovolemic shock	Lead colic, lead palsy and lead encephalopathy	Aanemia (less Hb), hypertension, kidney damage, miscarriages, disruption of nervous systems, brain damage, infertility, intellectual disorders	[26,27]

Compared to the organic form that mainly affects the central nervous system, inorganic mercury follows a non-uniform pattern of distribution, accumulating mainly in the kidneys; thereby, causing acute renal failure. Its impact on renal function is evaluated either by estimating glomerular function assessed mainly through the presence of high molecular weight proteins like albumin, transferrin, *etc.* or by tubular function assessed by low molecular weight proteins such as α1-microglobulin (α1-MG), β2-microglobulin (β2-MG), and retinol binding protein (RBP) in urine [28,29,30,31] (Table 2). Binding of mercury to sulfhydryl groups disrupts tubular enzymes such as *N*-acetyl-β-d-glucosaminidase (NAG) function, and its effect on sulfhydryl-containing enzymes is also used in assessment of renal tubular function [31]. A study by Li *et al.* (2013) reported evaluation of serum creatinine and blood urea nitrogen as an indicator for estimation of renal function or nephrotoxic effect of exposure to mercury [32]. Comparing binding affinity to different compounds, inorganic mercury shows higher binding affinity for endogenous thiol containing molecules such as glutathione and cysteine than to oxygen and nitrogen containing ligands [33]. An excess of glutathione increases the tendency of each mercuric ion to undergo coordinate complex formation with two glutathione molecules (2 glutathione: 1 mercuric ion) compared to 1:1 ratio for organic mercurial such as MeHg.

Through tubular microdissection studies, uptake and, as such, accumulation of inorganic mercury in the kidneys was found to occur mainly in the convoluted and straight segments of the proximal tubule [34,35]. There is evidence suggesting mercury–thiol conjugates of glutathione as the principal entity involved in the uptake of mercury at the proximal tubule of kidneys. Regarding its uptake, studies suggest involvement of different transporters operating at the luminal and basolateral membrane [10,33]. At the luminal surface, involvement of γ-glutamyltransferase (γ-GT) that catalyses the cleavage of γ-glutamylcysteine bond of the glutathione molecule performs an important role in the transport of mercury [33]. Pre-treatment with acivicin (inhibitor of γ-GT) significantly decreases luminal uptake and cellular accumulation of mercuric ions, thereby increasing urinary excretion of mercury in mice and rats [36,37]. Considering the presence of mercuric glutathione conjugates in the tubular lumen and the intimate relationship in the activity of γ-GT with the luminal uptake of mercuric ions by proximal tubular cells, transport of the by-products of γ-GT such as mercuric conjugate of cysteinylglycine appears quite possible. However, expression of the membrane bound dehydropeptidases (e.g., cysteinylglycinase) makes the rate of its transport low. As such, transport of mercury appears to occur through the luminal membrane as a conjugate of L-cysteine such as the dicysteinylmercury via one of the amino acid transport systems [38,39]. A study of Cannon *et al.* (2000), who reported increased uptake at the luminal surface of mercuric conjugates of cysteine, provided convincing evidence about the preference of its transport as a conjugate of cysteine rather than glutathione or cysteinylglycine [40]. Having similarity in structure to that of amino acid cysteine, it is postulated that molecular mimicry is one of the mechanisms involved in the luminal uptake of dicysteinylmercury [10,33].

In addition, there is sufficient evidence attributing 40%–60% of renal mercury burden to a basolateral mechanism through a renal organic anion transporter [41]. Mercuric conjugates of glutathione and/or cysteine are the primary entities transported across the basolateral membrane of proximal tubular cells by this same transport system. Taken together, transport of mercury to proximal tubular cells occurs through different transporters; at the luminal surface by amino acid transporters and at the site of the basolateral membrane through organic anion transporters 1 and 3 (Oat1 and Oat3) [42]. A study that reported abolishment of kidney injury in Oat1 knock-out mice helped in establishing it as a potent candidate involved in inducing HgCl_2_ mediated acute injury to kidney [43]. Besides this, another transporter, multidrug resistance-associated protein 2 (Mrp2), has also been associated with the elimination of mercury at the renal surface [44,45]. Although its presence has been reported in all proximal tubular cells, there lies heterogeneity in its expression along different segments of the proximal tubule.

#### 2.1.2. Mercury Induced Neurotoxicity

Neurotoxic effects are mainly associated with the organic form of mercury following its accumulation in the motor regions of brain and central nervous system (CNS). In nature, the majority of MeHg is contributed by the action of microorganisms in an aquatic ecosystem through biomethylation of inorganic mercury derived mainly from anthropogenic sources [46]. Possessing an enormous potential to undergo biomagnification, its accumulation in fishes renders communities highly vulnerable to its toxicity [19,47]. Based on the recommendations of an expert panel of the National Academy of Sciences, MeHg level exceeding 5 μg/L in whole blood and 1 μg/g in hair against the reference dose of 0.1 μg/kg bw/day is known to exert adverse health effects in humans [48]. Recently, Food and Agricultural Organization/World Health Organization Joint Expert Committee on Food Additives (JECFA) set a value of 1.3 μg/kg bw with reference to hair mercury concentration of 1.8 μg [49]. Oral fraction representing a major source through which humans are exposed, contributes significantly to its distribution following absorption (90%–95%) in the GI tract [50]. Despite scarcity of available data regarding speciation of MeHg that is absorbed from the GI tract, MeHg bound cysteinyl residues is generally believed to be the form that readily crosses the blood brain barrier (BBB) [51]. The complex formed between MeHg and cysteine is structurally similar to amino acid methionine, mimic specific amino acid transporter so as to gain entry into the central nervous system (CNS) [10,33]. Subsequent to its transport in the CNS, it undergoes a process of demethylation that leads to generation and as such accumulation of inorganic mercury. With poor penetration through the BBB, its accumulation in the CNS over time causes greater susceptibility to its toxicity, particularly during the initial stages of brain development [52,53]. Inside the brain, disturbance in the homeostasis of excitatory neurotransmitter, glutamate attributed to over-activation of *N*-methyl-d-aspartate (NMDA) type glutamate receptors, leads to increased Ca^2+^ influx into neurons [53,54]. Rapid increases in intracellular calcium level from extracellular calcium pools acts as a potent neurotoxin disturbing production of neurotransmitters, whereby creating serious imbalances in the development of the brain [55,56].

In addition to modulation in the transport activity of MeHg that affects production of neurotransmitters, studies related to molecular target and biochemical effects produced have also been reported using different experimental model systems. All these studies have established that MeHg exhibiting high affinity for sulfhydryl (thiol) and selenohydryl (selenol) groups besides causing impairment in protein function leads to depletion of intracellular antioxidants and causes inhibition of several important enzymes [57,58]. Glutathione (GSH), the most abundant thiol in mammals, is a prime target in mediating MeHg toxicity. Having high affinity for thiol and selenol groups, its binding to these groups led to interference in the activity of proteins such as thioredoxin (Trx), thioredoxin reductase (TrxR) and glutathione peroxidase (Gpx). By disrupting their activity, it affects the reduction of glutathione disulfide (GSSH), associated with the maintenance of the redox balance (GSH: GSSH ratio) of cells [59,60,61]. Interaction of MeHg with the thiol group of GSH increases excretion of MeHg-GSH conjugate complex. Studies performed in neuronal and glial primary cultures, isolated mitochondria from the mouse brain and non-neuronal cell lines, reported an intimate association between decrease in the GSH level and occurrence of oxidative stress [62,63,64]. Targeting thiol containing proteins of the respiratory chain along with other enzymes, it increases the production of ROS; thereby exacerbating damage to nucleophilic centers of mitochondria and other sub-cellular components [63,65]. Of the different pro-oxidant effects of MeHg, it is associated with increased levels of H_2_O_2_. In addition to a decrease in GSH level, increase in H_2_O_2_ levels is also attributed to its inhibitory effect of glutathione peroxidase (Gpx) either through capture of a selenol group located at its active site or by creating a selenium deficient like condition that affects synthesis of Gpx [59,66]. Consistent with this, increased generation of H_2_O_2_ that acts as a potent inhibitor of astrocyte glutamate uptake, has also shown association with glutamate homeostasis [67,68]. Taken together, disturbance in the oxidant/antioxidant balance in favor of the former represents the main event in mediating MeHg induced toxicity.

Of the two prevalent forms of organic mercury, ethylmercury (EtHg) gains entry into the body mainly as part of vaccines. Over the years, a large number of studies have been dedicated to sodium ethylmercury thiosalicylate, commonly referred to as Thimerosal. Developed in 1927, it is used as a preservative in pharmaceutical preparations, different ointments, cosmetics, and vaccines [18]. Although banned from use as a preservative, its use in the developing world still prevails in many childhood vaccines including tetanus toxoid (TT), Diphtheria-Tetanus-Pertussis (DTP), inactivated influenza vaccines, meningococcal meningitis vaccine and in several others [69]. As part of vaccines, it remains the main source of Hg exposure in children. During the pregnancy period, women are exposed through the use of inactivated influenza vaccines. It has been found that breast feeding also contributes to some extent to exposure of infants to substantial levels of mercury [18]. Having higher stability over EtHg, MeHg significantly affects fate of mercury and as such ranks highest than EtHg in terms of toxicity. EtHg that undergoes rapid conversion into inorganic mercury follows its path of toxicity, rather than the way MeHg induces its toxicity. Estimated half-lives (in days) for EtHg were 8.8 for blood, 10.7 for brain, 7.8 for heart, 7.7 for liver and 45.2 for kidneys [21]. It is to this, EtHg exposure through thimerosal-containing vaccines (TCV) in pediatric populations is mainly associated with renal and central nervous system toxicity. Compared to MeHg, conversion EtHg to inorganic forms helps body to get rid off its toxic effects.

### 2.2. Arsenic (As)

Arsenic, a naturally occurring metalloid, has ubiquitous distribution in the environment. Despite being the 20th most abundant element in the earth’s crust, it ranks highest on the list of hazardous substances toxic to public health [70]. Its existence as elemental, inorganic, and organic in large quantities all over the world makes it one of the most important metals, having adverse effects on the environment and human health [71]. Existing in more than 200 different mineral forms, its availability as arsenate (As^V^) accounts for approximately 60%, as sulphide or sulfosalt 20% and the remaining 20% in the form of arsenites, arsenides, oxides, silicates, and elemental arsenic [72,73]. Volcanic activity, weathering of rocks, geothermal waters, and forest fires constitute some of the natural sources of arsenic. In addition to pollution from natural sources, its applications in animal feed, glass and ceramics, herbicides, pesticides, wood preservatives, metallurgical operations and many others contribute to its anthropogenic pollution. Humans generally encounter arsenic by natural as well as manmade sources through soil, water, air, and food (Table 1) [72,74]. It is readily found in appreciable concentrations in food items having origin from the sea. Its mere presence in rice, a staple food crop worldwide makes its entry into the human body easier than other sources [6,75]. Through rice, it poses a greater risk to infants who mainly depend on rice for their meals (baby foods). As per World Health Organization (WHO) guidelines, a safer limit of 200 μg/kg was established for white rice and a maximum of 400 μg/kg for brown rice [75]. As a group I carcinogen, its contamination of drinking water is a serious environmental calamity worldwide [76]. Toxicity associated with water contaminated with arsenic has been reported from different countries including Bangladesh, India, China, *etc.* [76,77]. It is estimated that around 200 million people are exposed predominantly through drinking water, having its concentration greater than the prescribed limit [78].

In nature, soluble arsenic exists in two common oxidation states, arsenate (As^V^) and arsenite (As^III^) present as the oxyanions arsenate (AsO_4_^3^–) and arsenite (As(OH)_3_), respectively. Following ingestion, it is readily absorbed (>90%) by the gastrointestinal tract. Their toxicities and as such cellular damage vary with respect to their valence states. Transport of As^V^ into enterocytes occurs by means of high-affinity phosphate transporters while that of As^III^ involves a wide variety of transporters such as glucose transporters (GLUT2, GLUT5), organic anion transporting polypeptides (OATPB), and aquaporins (AQP3 and AQP10) [79,80,81]. Difference in their toxicities along with their biological affects arises with respect to their uptake and as such accumulation in the cellular system; pentavalent arsenicals (As^V^) taken up less efficiently show a lower rate of accumulation than trivalent species (As^III^) [82]. Having greater uptake and high affinity for sulhydryl (–SH) groups of proteins and enzymes, As^III^ is considered more toxic than its counterpart, As^V^ [71,83]. However, owing to its structural similarity to phosphate, As^V^ exerts its toxicity through replacement of phosphate in different chemical reactions. As part of their toxicity, As^V^ replaces the stable phosphodiester bond in ATP, thereby resulting in uncoupling of oxidative phosphorylation events and subsequent depletion of ATP stores, while depletion of the intermediate of Krebs cycle by As^III^ results in the exhaustion of cellular energy via inhibition of cellular respiration [84].

Humans generally encounter arsenic by natural means, industrial sources, or from unintended sources. Adhering to WHO safety guidelines, the maximum permissible level of arsenic in drinking water is 10 μg/L [85]. Due to their significant toxicity, arsenic compounds are associated with a wide range of health problems ranging from gastrointestinal disturbance to development of neoplasms, particularly of the skin, liver, kidney, and lymphatic cancer [86]. As part of their toxicity, arsenic induced superoxide (O_2_^•**−**^) is known to disrupt various cell signalling pathways. Both superoxide (O_2_^•**−**^) and the subsequently generated H_2_O_2_ and ^•^OH interact with biological macromolecules, leading to DNA damage, lipid peroxidation and alteration of the levels of antioxidant enzymes such as superoxide dismutase (SOD) and catalase (CAT) [86]. Though short term exposure to low level arsenic causes reduction in the production of erythrocytes and leukocytes, damage to blood vessels, nausea and vomiting, abnormal heartbeat, and pricking sensations in the hands and legs, its exposure for long time periods often leads to skin lesions, peripheral vascular disease, pulmonary disease and cardiovascular diseases, neurological problems, diabetes mellitus and certain types of cancers [87,88]. Chronic arsenicosis results in many irreversible changes in vital organs and a higher mortality rate. Despite the magnitude of this potentially lethal toxicity, there is no effective treatment for this disease.

Arsenic, a protoplastic poison, primarily affects the sulfhydryl group of cells, leading to malfunctioning of cell respiration, cell enzymes, and mitosis [89,90]. Through the involvement of bacteria, biotransformation of arsenicled to production of methylated compounds such as monomethylarsonic acid (MMA) and dimethylarsinic acid (DMA). Within cells, metabolism of arsenic specifically occurs through a series of methylation reactions catalysed by methyltransferases [91]. For their activity these enzymes depend on a methyl group donor, *S*-adenosylmethionine (SAM), and presence of a reductant such as Glutathione (GSH) or TR/Trx/NADPH system [92,93]. Although liver is the primary site for methylation, expression of folate dependent methyltransferases has also been reported in lung, heart, kidney, and bladder tissues [91]. Several reports have suggested that binding of arsenic species to GSH increases its elimination from the body. As such, biomethylation serves as a detoxification process, and end products (methylated inorganic arsenic such as MMA^V^ and DMA^V^ excreted through urine) provide bioindications of chronic arsenic exposure (Table 2). As part of detoxification strategies operating in the cellular background, metabolically generated methylated species have been found to be even more toxic than their parent species. In comparison of toxicities between As^III^ and its monomethylated derivative, monomethylarsonous acid (MMA^III^), its derivative, exhibited higher toxicity due to higher affinity for sulfhydryl (–SH) groups [94]. Rather than detoxification, it appears to function as activation pathways through which enhancement of their toxicities is achieved. Toxicities of metabolically generated methylated species vary with respect to their extent of undergoing methylation. Based on findings of different cytotoxicity studies performed under both *in vitro* and *in vivo* conditions, relative toxicities of arsenic metabolites follows the order; MMA^III^ > DMA^III^ > As^III^ > As^V^ > MMA^V^ > DMA^V^ [83,94,95]. On one side where GSH acts as a flag that increases its excretion (60-70%) at the surface of kidneys or through bile (80%–90%); methylation at the same time led to increase in its cytolethality. Transport of arsenic bound to GSH [As-(GS)_3_ or MMA-(GS)_2_] from liver into bile occurs through the involvement of ATP binding cassette (ABC) transporter, MRP2 and to blood through MRP1 [79,80,81]. Compared to *p*-glycoproteins (PGP) that transport GSH conjugated arsenicals, efflux of As^III^, MMA^III^, MMA^V^, and DMA^V^ occurs through aquaporin isozyme 9 (AQP9) [81]. In addition, transport of As^III^ and MMA^III^ across the cellular membrane occurs through glucose transporter, GLUT2 [80]. In short, biomethylation serves as a detoxification process, and presence of its end products (methylated inorganic arsenic such as MMA^III^, DMA^III^, *etc.*) in urine acts as bio-indicators of chronic arsenic exposure.

### 2.3. Lead (Pb)

Lead (Pb) is one of the most abundant natural substances on earth. Owing to its physical properties including low melting point and high malleability, it has widespread industrial use. In terms of usage, it ranks fifth on the list of metals [96]. Its use is associated with more than 900 industries, including mining, smelting, refining, battery manufacturing, and so on [96,97]. In addition to industry, it has applications in fertilizers and pesticide used for agriculture purposes, and in improving the octane rating of gasoline in vehicular traffic systems [98]. As a result of rapid industrialization, increase in the effluent discharge from industrial units located in close proximity to rivers has resulted in an increase in its amount in water bodies [96]. Along with this, application of sewage sludge directly or as part of irrigation from contaminated water bodies, as an exhaust product of leaded gasoline due to increased traffic activities in urban settings and increased use as part of fertilizers and pesticide for agricultural purposes has resulted in the pollution of soils, which has had a serious environmental impact [99,100]. Together, these (agricultural, industrial, and municipal) activities have resulted in the contamination of groundwater resources [101]. In short, its abundance and widespread usage makes it a well-recognized environmental and occupational toxicant, particularly in the urban environment.

Being unnecessary for the human body, the prescribed limit for drinking water set by WHO is 0.01 mg/L (10 μg/L) [96]. Indicated as a persistent pollutant, humans are exposed mainly through occupational settings. As such, working populations are more prone to the risk of lead toxicity. The prevalent method for assessing its exposure and, as such, its toxicity is to check the blood Pb level (BLL). However, on the recommendations of the Advisory Committee on Childhood Lead Poisoning Prevention (ACCLPP), the term BLL has now been replaced by “BLL reference level” [102]. Over the years, BLL reference level set by the Centers for Disease Control (CDC) has changed from 60 μg/dL in 1960s to 5 μg/dL for children in the age group of 1–5 years [102]. Acceptable BLL reference level for the working population ranges between 30 and 49.9 μg/dL [103]. However, BLL reference level of <49 μg/dL was associated with impaired cognitive, behavioural, and motor development in children and, along with this, hypertension, nephropathy, and impaired fertility in adults [104]. Following exposure, blood lead level (BLL) greater than 70 μg/dL and 100 μg/dL was found associated with significant toxicity in children and adults, respectively [105]. Adults with BLL less than 70 μg/dL generally do not require chelation therapy. However, chelation therapy with succimer is advisable as a treatment option for children with BLL between 45 and 69 μg/dL [106]. Some reports have suggested that low BLL reference level that remains a point of controversy for chelation therapy may lead to permanent neurologic sequelae and even to death [107]. While recognizing that even a small amount of it is harmful, it appears that reducing its toxicity in humans with use of chelating agents is not possible. Although exposure mitigation strategies imposed by government or through public health interventions have led to a significant decline in the symptomatic poisoning of the human population, childhood lead poisoning is still regarded as an important public health concern worldwide.

Lead, a potent occupational toxicant with widespread use, is of high concern owing to extensive contamination of the environment that has caused severe health problems in many parts of the world. Representing a stable pollutant; clinical manifestations of its toxicity range from subclinical and subtle features to life-threatening complications [104]. Acute exposure can cause loss of appetite, headache, hypertension, abdominal pain, renal dysfunction, fatigue, sleeplessness, arthritis, hallucinations, and vertigo, while chronic exposure can result in intellectual disability, birth defects, psychosis, autism, allergies, dyslexia, weight loss, hyperactivity, paralysis, muscular weakness, brain damage, kidney damage, and even death (Table 1) [108]. Although toxicity of lead from industrial settings has been relatively controlled, it remains a pervasive toxicant worldwide [109]. Despite the fact that humans are exposed through multiple sources, inhalation through airborne dusts containing lead particles and ingestion through food or water contaminated by Pb are considered the most probable routes of exposure. Although its absorption through intestines depends on its physical and chemical properties, age of individuals was found to have a pronounced effect on its toxicity [96]. Compared to adults, children are very prone to its toxicity due to play behaviour and increased hand to mouth activities [110]. It is reported in the literature that children who show a rapid growth course showed increased intestinal absorption of lead compared with adults as this capacity significantly decreases with age [111]. After absorption at the intestinal interface, it is transferred as part of blood (B-Pb) to soft tissues (liver, kidney, lungs, *etc.*) and mineralizing tissues (bone and teeth). Although blood contributes only a small fraction to total body burden of Pb, it performs an important role in its distribution throughout the body, thereby making it available to tissues as well as for excretion by the kidneys. Owing to its half-life ranging from 30 days in blood to decades in bone, most of the body’s burden of Pb is attributed to its presence in bone [112]. Its accumulation over time is considered a good indicator of its toxicity; with its presence in blood representing a recent exposure while its presence in bone reflects long term burden of the body.

Lead toxicity is a particularly insidious hazard with the potential to cause irreversible health effects. In evaluation of its toxicity in humans, it was found that bone to blood mobilization increases during pregnancy, lactation, physiological stress, chronic disease, along with advanced age [113]. Its release back into the bloodstream, particularly during times of calcium stress in the pregnancy period, makes the developing foetus more prone to its toxicity through mobilisation as part of the blood supply and after birth through lactation (breast feeding) of the infant. Elevated levels in pregnant women often lead to preterm labor, miscarriages, spontaneous abortion or still births and low birth weight children [113]. In the blood, it produces its effect by interfering with the biosynthesis of heme [114]. Having high affinity for thiol (–SH) groups, it affects the functioning of δ-aminolevulinic acid dehydratase (δ-ALAD), catalyzing the conversion of two molecules of δ-aminolevulinic acid (δ-ALA) to porphobilinogen (PBG) [114,115]. Inhibition of the activity of δ-ALAD causes accumulation of δ-ALA in blood. In addition, it prevents incorporation of Fe^2+^ into the protoporphyrin molecule, accompanied by binding of zinc (Zn^2+^) rather than Fe^2+^ into the protoporphyrin ring, thereby producing zinc protoporphyrin (ZPP) [116]. Presence of δ-ALA or ZPP in blood or in urine is considered a potent marker for early biogenic effect of lead in humans (Table 2). On treating rats with δ-ALA, increased levels of 8-oxo-7,8-dihydro-2′-deoxyguanosine, and 5-hydroxy-2′-deoxycytidine, give an indication of δ-ALA induced DNA damage through the involvement of ·OH [117,118]. There are also reports suggesting a genotoxic effect of δ-ALA through its oxidant product, 4,5-dioxovaleric acid, which acts as a strong alkylating agent [117,118]. Alternatively, through binding to –SH groups of both reduced (GSH) and oxidised (GSSH) glutathione, it disturbs the GSH/GSSG balance and, as such, renders cells more prone to oxidative damage [115]. Antioxidants present in the cells, such as glutathione (reduced), give their reducing equivalents to ROS in order to make them stable and as such protect the cell from free radicals such as H_2_O_2_. However, under the influence of lead, the level of ROS increases while that of antioxidants decreases. As part of the antioxidant enzymes such as GPx, CAT, and SOD, which depend on trace elements and prosthetic groups to accomplish the enzymatic detoxification of ROS, they are also potent targets to Pb toxicity [115]. Along with hampering the activity of antioxidant enzymes, depletion of antioxidants and protein bound sulfhydryl groups have been implicated in Pb induced oxidative damage.

Capable of causing oxidative damage, polyunsaturated fatty acids having a larger number of double bonds in their structure are prone to damage by oxidative stress. As such, transfer of Pb makes the erythrocyte membrane more vulnerable to oxidative damage than other tissues [119]. Disturbance in lipid composition through generation of hydroxyl (·OH) and peroxynitrite as part of oxidative stress is known for its cardiovascular effects [120,121]. Oxidation of membrane lipids through its interaction with Pb results in altered membrane integrity, permeability, and function. Of the different risk factors associated with cardiovascular disease, an increase in the level of total cholesterol, triglycerides, and elevated lipoprotein content attributed to exposure from Pb compounds is manifested with increased risk of cardiovascular disease [121]. Changes in polyunsaturated fatty acids, induction of lipid peroxidation and disturbance of membranous enzymes are common mechanisms through which lead contributes to cardiovascular complications. Through studies, it is now well established that exposure to low levels of Pb causes elevation of blood pressure, and, if prolonged, can promote development of arterial hypertension [121,122]. Taking different studies into consideration, it is now speculated that the mechanism behind genesis of Pb related arterial hypertension arises either directly through its vaso-constrictive effect or by disturbance in the activity of endothelial derived relaxing factors [123,124,125,126].

By regulating the blood volume and vascular tone, kidneys play a major role in their effect of regulating the blood pressure. Higher lead levels represent an independent risk factor for arterial hypertension; therefore, any alteration to kidney function that causes constriction of blood vessels, thereby raising blood pressure, contributes to severity of cardiovascular complications [123,126]. Inside the body, it interferes with a number of bodily functions, thereby affecting most organs. Compared to inorganic lead that is not metabolized by liver, ingested organic forms are absorbed and, as such, metabolized by the liver. Lead is known to stimulate synthesis of lipid in different organs, particularly in liver [127]. It has an ability to mimic and replace essential cations such as Ca^2+^ and Mg^2+^, which are involved in various biological processes including cell adhesion, intra- and inter-cellular signalling, apoptosis, ionic transportation, protein folding, enzyme regulation, and release of neurotransmitters [128,129]. At picomolar concentrations, substitution of lead for calcium affects activity of protein kinase C, which regulates neural excitation and memory storage [130]. Though humans are exposed to lead primarily through environmental and domestic sources, it is possible to reduce the risk associated with toxicity by taking proper precautionary measures.

## 3. Cytotoxic Mechanisms of Heavy Metals

**Figure 1:**
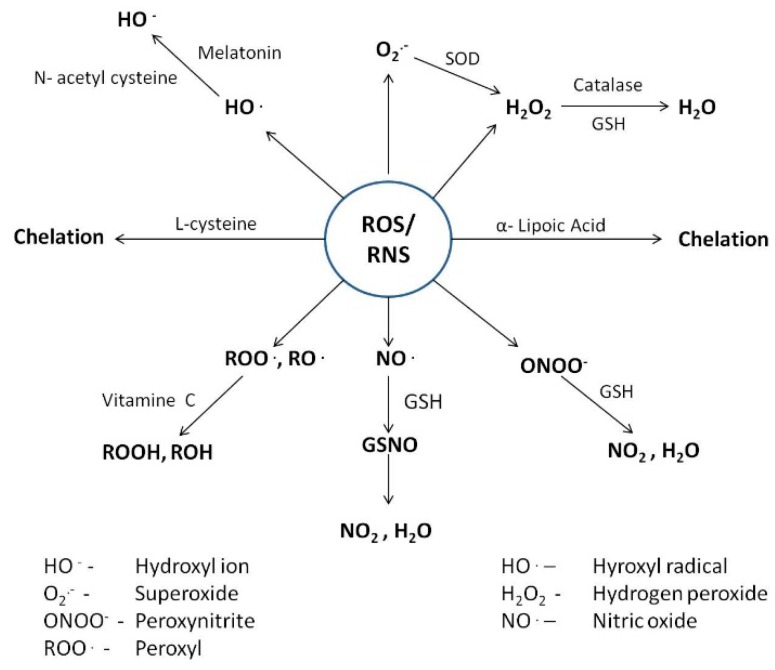
Modes of action of different antioxidants in mitigating the toxic effects imposed by metals.

Heavy metal induced toxicity has been studied extensively and reported by various workers. Having the potential to produce highly reactive chemical entities such as free radicals, heavy metals are known to cause oxidation of sulfhydryl groups of proteins, depletion of protein, DNA damage, lipid peroxidation, and several other effects. The underlying factors making the greatest contribution to toxicity for different metals involves generation of reactive oxygen (ROS) and nitrogen (RNS) species that disturb cell redox systems. ROS that are distinguished by their high chemical reactivity, include free radicals such as superoxide (O_2_^•**−**^), hydroxyl (OH^•^), peroxyl (RO_2_^•^) and alkoxyl (RO^•^), as well as certain non-radicals such as peroxynitrite (ONOO^−^) and H_2_O_2_, which are either oxidizing agents or get easily converted to radicals (Figure 1).

O_2_ + e^−^ → O_2_^•**−**^

2 O_2_^•**−**^ + 2H^+^ → H_2_O_2_ + O_2_

H_2_O_2_ → 2 ^•^OH

Intracellular generation of superoxide anion (O_2_^•−^) primarily occurs non-enzymatically through the intervention of redox components such as semi-ubiquinone (a component of the mitochondrial electron transport chain) [128,129,130,131], or via the intervention of enzymes such as NADPH-oxidase (NOX) [132], xanthine-oxidase or auto-oxidation reactions [133,134]. Superoxide anion (O_2_^•−^) acts as a mild reactant under physiological conditions, with poor ability to cross the biological membranes. Upon interaction with nitric oxide (NO), production of peroxynitrite (ONOO**^−^**) transforms superoxide into very reactive intermediates such as hydroxyl radical (^•^OH), which have a very short half-life [135].

NO + O_2_^•**−**^ → ONOO**^−^** + H^+^ → ^•^OH + ^•^NO_2_

Through the involvement of nitric oxide synthase isozymes like endothelial nitric oxide synthase and (eNOS) mitochondrial nitric oxide synthase (mtNOS), generation of nitric oxide occurs via conversion of l-arginine to citrulline. NO^•^ has been shown to have greater stability in oxygen deprived environments. Because of its amphipathic nature, NO^•^ easily diffuses through the cytoplasm and plasma membranes. Upon interacting with superoxide anion, NO generates peroxynitrite (ONOO**^−^**) [136]. An increase in ROS/RNS production or decrease in ROS-scavenging activity that arises as a result of exogenous stimuli has been found to alter cellular functions through direct modifications of biomolecules and/or by aberrant stimulation/suppression of certain signalling pathways affecting growth factor receptors.

### 3.1. ROS/RNS and Protein Destruction

Proteins that have broad spectrum functionalities, ranging from structural proteins (myosin, tropomyosin) to glycolytic enzymes (enolase) to regulatory enzymes, that have a role to play in synthesis of protein (elongation factors), its folding (heat shock proteins), degradation (ubiquitin thiolesterases) and to defence as an antioxidant (thioredoxin), have been found to be greatly affected by ROS/RNS species [137]. Metals interfere with the biological activity of proteins through diverse mechanisms, they may; (1) displace essential metal ions; (2) bind to free thiol (–SH) groups of proteins; (3) catalyze oxidation of amino acid side chains; (4) interfere with the folding of protein into 3D structure; and (5) in some cases prevent their refolding [138]. Binding of exogenous metals to protein ligands by displacing physiological metals from their natural carriers causes disruption of cell physiology. Binding of metal cations to adventitious ligands that result in steric rearrangements also impairs physiological function of cellular proteins. More importantly, hydroxyl (^•^OH) radical generated through fenton reaction and powerful oxidant peroxynitrite (ONOO**^−^**) that subsequently generates nitrite (^•^NO_2_) and hydroxyl ions are common reactive species that target proteins. Damage to proteins caused by different metals mainly involves loss of histidine residues, carbonyl group attachment and bityrosine cross links in addition to generation of carbon centered alkyl radical (R^•^), alkoxyl (RO^•^) and peroxyl (ROO^•^) radicals [139].

R–H + ^•^OH → R^•^ + HOH

ROOH + Fe^2+^ → RO^•^ + **^−^**OH + Fe^3+^

R^•^ + O_2_ → ROO^•^

In addition to amino acids (histidine, arginine, proline, cysteine, *etc*.) that have side chains vulnerable to attack by ROS and RNS, sulphur containing amino acids are also susceptible to reversible oxidation of sulfhydryl groups. These include intermolecular (P1-S-S-P2) or intramolecular (P1-S-S-P1) cross linkages and glutathionylation by disulphide exchange (PSH + GSSG → PSSG + GSH) catalyzed by thiol transferase or irreversible oxidation such as nitrosylation of sulfhydryl groups of cysteine and methionine by peroxynitrite (ONOO**^−^**) [140,141]. By interfering with the folding and refolding process of native proteins, they disturb protein homeostasis by causing their aggregation in living cells [142,143]. By enhancing the aggregation propensity of disease associated proteins, they are believed to contribute to progression of certain neurodegenerative diseases. Detailed insights into the metal induced oxidative damage to proteins are provided by Reyes *et al.* (2013) and Tamas *et al.* (2014) in their studies [144,145].

### 3.2. ROS/RNS and Lipid Peroxidation

Lipids are most sensitive to oxidative modification by ROS. In aerobic organisms, membrane phospholipids are continually subjected to oxidation by both endogenous and exogenous sources. Lipid peroxidation has been implicated as a major mechanism in the generation of diseases and disorders such as cancer, cardiovascular and neurodegenerative diseases that function through alteration of the integrity, fluidity, and permeability of the membranes [146] and references cited therein]. Being chemically reactive and potentially toxic, products of lipid peroxidation such as malondialdehyde (MDA), 4-hydroxy-2-nonenal (HNE) and acrolein (markers of lipid oxidation) exert their effects by modifying essential molecules such as proteins and DNA bases [147,148,149,150]. Lipid peroxidation is used as a standard for metal-induced oxidative stress, particularly in hepatocytes, testes, and liver mitochondria of rats [148,149,151]. Under pathological conditions such as atherosclerosis and ischemia, oxidation by means of ROS or other free radicals of polyunsaturated fatty acids is thought to be exacerbated by the presence of divalent metal ions. Upon interaction with copper, chromium, nickel, and cadmium, metals and their chelate complexes are believed to play an active role in lipid peroxidation and in the promotion of carcinogenesis [152,153]. However, interaction of lipid peroxyl radicals with lipid radicals that leads to formation of non-radical species causes termination of lipid peroxidation.

LH + R^•^ or ^•^OH → L^•^ + RH or HOH

L^•^ + O_2_ → LOO^•^

LOO^•^ + LH → LOOH + L^•^

LOOH → LO^•^ + LOO^•^

### 3.3. ROS/RNS and Nucleic Acid Destabilization

Hydroxyl (^•^OH) radical, the most powerful oxidising radical, is responsible for causing damage to most biomolecules. In nucleic acids, both nitrogenous bases and sugars are highly susceptible to attack by electrophiles, particularly hydroxyl (^•^OH) radical. Upon interaction with the sugar moiety of DNA, ^•^OH radical cause abstraction of H from sugar, thereby resulting in strand breaks [154]. Although double bonds within nitrogenous bases are prime targets for ^•^OH attack, oxidation reactions are primarily centred at C-5 and C-6 of pyrimidines and at C-4, C-5, and C-8 of purines [155]. Reactions of ^•^OH radical with pyrimidines and purines result in multiple adduct products in DNA [154]. Pyrimidine adduct (C5–OH and C6–OH) radicals differ in terms of their redox properties, with C5–OH being reducing and C6–OH being oxidising. C4–OH and C5–OH purines adduct radicals dehydrate and, as such, get converted to oxidizing purine (–H)^•^ radical, which, upon reduction and protonation, reconstitute purines [156]. Purine-adduct (C4-OH) radicals are primarily oxidising; while C5–OH and C8–OH adducts possess reducing properties. Based on their redox properties, reaction partners and the presence or absence of oxygen, pyrimidine as well as purine radicals undergo oxidation or reduction to yield numerous products such as cytosine glycol and thymine glycol [157,158]. All these adducts that undergo different transitions and transversions are represented as premutagenic lesions. Another DNA oxidant is peroxynitrite (ONO_2_^−^). Exposure of DNA to ONO_2_^−^ that results in strand breaks and base oxidation products shows 8-nitro-deoxyguanosine as the major product of reaction [159]. This adduct has a tendency to depurinate. Besides that, it has also been found to inhibit DNA repair enzymes, such as Formamido Pyrmidine (FAPY) DNA glycosylase, associated with the removal of 7,8-dihydro-8-oxoguanine (abbreviated as 8-oxo-G or 8-OH-G). Together, it points towards existence of synergism between generation of peroxynitrite and ability to inhibit the repair of this damage [160,161]. 8-hydroxyguanine (8-OHG) and 8-hydroxydeoxyguanosine (8-OHdG), the most frequent base lesions, are used as a marker of oxidative DNA damage [162]. Due to its premutagenic ability, 8-oxo-guanine is well known for its contribution to human diseases.

Cells have repair systems that check DNA and remove oxidative DNA lesions associated with mutagenesis and cytotoxicity. Under normal circumstances, oxidized base lesions of DNA are removed by two types of repair systems, base excision repair (BER), which involves removal of single lesions following glycosylase action, and nucleotide excision repair (NER). Of the two repair systems, base excision repair (BER) being more specific in removing lesion-containing oligonucleotides, predominates in the repair of oxidative DNA damage. Compared to pyrimidine lesions, the rate at which purine lesions are repaired is slow [163]. As most of the mutations are acquired during the replicative phase, the repair system repairs damage well before the cell reaches the replication stage [164]. As such, susceptibility of DNA to oxidative modifications by ROS and RNS is lower than that of proteins and lipids. However, when ROS/RNS levels are high, biological consequences are often deleterious. When compared to moderate levels of DNA damage that trigger cell cycle arrest and initiate DNA-repair processes to ensure DNA integrity, excessive damage in DNA repair can induce apoptosis [165].

## 4. Counteractive Antioxidant Defence

Under physiological conditions, redox homeostasis (an intricate balance between rate of generation of ROS, free radicals and other reactive intermediates and their removal) is primarily controlled by cellular reductants, essential mineral ions and enzymatic antioxidants such as catalase, superoxide dismutase, and glutathione peroxidase. Antioxidants that act at different levels of the oxidative sequence act by depleting molecular oxygen, quenching singlet (^1^O_2_) oxygen species, trapping aggressive reactive species such as superoxide radical (O_2_^•**−**^) and hydrogen peroxide (H_2_O_2_), scavenging chain initiation reactants such as hydroxyl (^•^OH), alkoxyl (RO^•^) or peroxyl (ROO^•^) radicals, and breaking the chain reaction sequence by decomposing products of oxidation to non-radical species so as to prevent hydrogen abstraction from different substrates.

R^•^/RO^•^/ROO^•^ + A–H → A^•^ + RH/ROH/ROOH

RO^•^/ROO^•^ + A^•^ → ROA/ROOA

L^•^/LO^•^/LOO^•^ + A–H → A^•^ + LH/LOH/LOOH

Maintaining the delicate balance between reactive oxygen species (ROS) and reactive nitrogen species (RNS) production and their removal by cellular reductants, antioxidant enzymes and small molecular weight antioxidants involves complex mechanisms. Dysfunction of any of these mechanisms due to exogenous stimuli or endogenous metabolic alteration often leads to an increase in intracellular ROS levels, which is associated with the generation of oxidative stress. The following section deals with the natural redox systems of the body, particularly mechanistic processes through which their antioxidant activity mitigates the toxicity imposed by heavy metals.

### 4.1. Cellular reductants

#### 4.1.1. Glutathione

Glutathione, an intracellular multifunctional non-enzymatic antioxidant known for its role as a major redox (thiol-disulphide) buffer of the cell, is abundant in cytosol (1–11 mM), nuclei (3–15 mM), and mitochondria (5–11 mM) [166]. Inside the cell, it exists in two forms, reduced glutathione (GSH) and oxidised glutathione disulphide (GSSG). GSH is a low molecular weight tripeptide (γ-glutamylcysteinyl-glycine) synthesised in the liver from amino acid precursors (l-cysteine, l-glutamate, and glycine) via a complete pathway starting with methionine, and progresses through homocysteine and cysteine to glutathione [167]. In mammalian systems, l-cysteine acts as an important line of defence against oxidative stress. Specifically, it is believed to perform a series of important physiological and metabolic functions including detoxification of free radicals, metals, and other electrophilic compounds [168].

R^•^/RO^•^/ROO^•^ + 2 GS–H_(reduced)_ → GSSG_(oxidised)_ + RH/ROH/ROOH

l-cysteine has been found to increase the antioxidant capacity of mitochondria by protecting it against the oxidative burst (H_2_O_2_, singlet oxygen, hydroxyl radicals, and lipid peroxides) generated by mercury [169]. However, under deficient or reduced expression of enzymes necessary for glutathione synthesis, l-cysteine is imported from cytosol. Reactions catalyzed by glutathione peroxidase that lead to depletion of reduced GSH and subsequent increase in the intracellular levels of oxidised glutathione (GSSG) are accompanied by reduced reconversion of GSSG to GSH by glutathione reductase, along with a decrease in the export of GSSG from the cell.

GSH has the ability to form complexes with different metals via non-enzymatic reactions (Table 2). Stabilization of metals attached to the sulfhydryl group of glutathione increases if it becomes coordinated to other binding sites present within the tripeptide. Metals have been found to form stable complexes if they combine with sulfhydryl groups in a 1:2 ratio (i.e., each metal ion coordinating with two molecules of glutathione via a sulphur atom on the cysteinyl residue of the glutathione) [47,168].

GS-Hg-SG (Mercury-glutathione complex)

Soon after the formation of mercury-glutathione conjugates in hepatocytes, they enter systemic circulation, which results in their delivery to the kidneys. In plasma, labile bonding between mercuric ions and thiol containing molecules results in a rapid decrease of plasma mercury burden with a concurrent increase in the uptake of mercuric ions by kidneys [24]. Inside the kidneys, GSH acts both as a carrier and antioxidant, performing a complex role in the regulation by GSH of renal cellular disposition, and therefore, the cytotoxicity caused by Hg^2+^ [170]. By preventing binding of Hg^2+^ to other essential cellular thiols, GSH protects renal cells from Hg^2+^ induced cellular injury, while also enhancing renal cellular accumulation of mercury as conjugates with extracellular glutathione rather than as free Hg^2+^ ions [33].

**Table 2:** Mechanistic insight into metal toxicity and mode of action of antioxidants and their health benefits in overcoming the deleterious effect of metals.

Metal	Mechanism of toxicity	Biomarkers of Toxicity	Antioxidants	Mechanism of action	Health effects	Ref.
Mercury Arsenic Lead	Oxidative and nitrative stress, alteration of thiol dependent pathways, depletion of intracellular antioxidants, binding to specific location and dislocation of essential ion, damage to macromolecules, inhibition of repair machinery, chromosomal abnormalities and altered gene expression, binding to –SH group and inhibition of enzymatic activity, membrane damage, inhibition of oxidative phosphorylation, inhibition of heme biosynthesis, disruption of protein structure, hypertension	Malondialdehyde (MDA), 8-OH-2-OxoG, Hg-GSH, albumin, transferrin, α1-microglobulin (α1-MG), β2-microglobulin (β2-MG), retinol binding protein (RBP), enhanced deposition in hair, bones and soft tissues, lipid peroxides, methylated products of arsenic (MMA^V^, DMA^V^), increased B-Pb level, increased disposal of δ-ALA and ZPP	Endogenous thiols (GSH, l-Cys, NAC, Taurine, Melatonin)	Scavenging of free radicals, interrupt radical chain reactions, formation of stable complexes with metals	Reduces metal availability, decreases damage to cell organs and biological macromolecules, Promotes detoxification	[28,29,30,31,59,60,61,86,96,114,115,116,138,139,140,141,147,148,149,150,162,163,164,171,172,173,174,175,176,177,178,179]
			Minerals (Se, Fe, Cu, Zn)	Competes with intestinal absorption, decreases replacement of essential ions, formation of insoluble metal-mineral complexes, induces production of metal binding proteins (MTs)	decreases GI absorption and as such its distribution, prevents redistribution and accumulation in tissues, reduces metal availability thereby decreases toxicity, Stabilizes cell membranes, decreases damage to biological macromolecules, decreases teratogenic toxicity	
			Enzymatic (SOD, GPx CAT)	Neutralize free radicals and as such attenuates oxidative damage	Protects cell organs and biological macromolecules, Stabilizes cell membranes	
			Vitamins (α-LA, Vit C, Carotenoids)	Scavenging of free radicals, decrease in cellular oxidative stress	Reduces plasma to lipid peroxidation, decreases risk of having stroke, reduces incidences of chronic and degenerative diseases, reduces sperm ROS generation and prevents loss of motility and oocyte penetration	

#### 4.1.2. l-cysteine and *N*-acetyl Cysteine

l-cysteine, a non-essential hydrophilic amino acid, possesses a thiol side chain that makes it highly reactive and, therefore, imparting it with biological properties. Dietary cysteine is primarily introduced to the body as the breakdown product of ingested proteins and peptides. Dipeptide cystine, which acts as the main extracellular source of intracellular cysteine, competes with glutamate for transport into the cell [180]. l-cysteine acts as an important component of a large number of enzymes and proteins. Biosynthesis of cysteine follows a trans-sulfuration pathway that starts with methionine and flows through homocysteine, which together with serine forms cystathionin and ultimately cysteine. L-cysteine possesses strong antioxidant properties due to the presence of a thiol group that easily undergoes redox reactions. l-cysteine has high affinity for mercury and other metals (copper, lead, cadmium, *etc.*), allowing their excretion from the body. In short, it is directly involved in the detoxification of heavy metals from the body of living organisms.

Cys-Hg-Cys (dicysteinyl mercury)

*N*-acetylcysteine (NAC) is the acetylated derivative of l-cysteine. Addition of the acetyl group speeds up the absorption and distribution of orally ingested cysteine and protects it against oxidative and metabolic processes [181]. The effectiveness of NAC is primarily attributed to its ability to reduce extracellular cystine to cysteine and its being a source of sulfhydryl groups. The antioxidant activity of NAC is attributed to its ability to maintain intracellular GSH levels, which is associated with scavenging ROS, as well as its ability to react with biological macromolecules and, as such, inactivate enzymes, lipid peroxidation, and DNA damage (Table 2). In addition to stimulating glutathione synthesis, NAC enhances glutathione-*S*-transferase activity and promotes liver detoxification by inhibiting xenobiotic biotransformation [182,183]. Despite being beneficial in reducing toxicity, both GSH and NAC conjugates of mercury possess a high risk of redistribution of mercury to the body by assisting in the uptake mechanisms at the surface of kidneys [42]. As both GSH and NAC conjugates of mercury are potentially transportable species, they contribute little to the elimination of different mercury species.

#### 4.1.3. Taurine

Taurine (2-aminoethanesulfonic acid) is a semi essential amino acid ubiquitous in the animal kingdom, but rarely found in the plant world. Intracellular concentration of taurine ranges between 5 and 20 μmol/g weight of tissues such as heart, brain, *etc.* [184,185]. Under normal circumstances, the majority of intracellular taurine is synthesized in liver from methionine and cysteine via cysteine sulfinic acid decarboxylase (CSDI). Owing to enzymatic immaturity, taurine, which probably acts as an essential amino acid, has limited capacity for its synthesis and relative inability to be conserved in the case of neonates [186,187]. As such, deficiency in neonates that appears to have a deleterious effect on the developing brain and retina requires continuous supplementation through parenteral nutrition [188,189]. Being hydrophilic, taurine provided as a dietary supplement is poorly absorbed and does not diffuse readily across membranes. Therefore, extremely high doses (typically > 3 g/day) are required to achieve any clinical efficacy. The presence of taurine transporters on the surface of many cells helps maintain intracellular taurine at concentrations significantly higher than extracellular levels. Besides being beneficial to the prevention of neurodegeneration in the elderly [190], dietary supplementation of taurine can reduce the risk of a number of diseases, including diabetes [191], inflammatory bowel disease [192], and cardiovascular disease [193].

Broad functional diversity of taurine contributes to its beneficial actions that include reactive-radical scavenging, membrane stabilisation, osmoregulation, and modulation of intracellular calcium homeostasis, neuroprotection, and neurotransmission [184,185] (Table 2). Under *in vitro* conditions, taurine plays a protective role against neurodegeneration by reducing the toxic effects of metals such as lead [194,195]. In relation to the adverse effects on taurine levels, metals such as lead are known for causing a significant decrease in the plasma taurine concentrations, while nickel diminishes the protection of membranes afforded by taurine [196,197].

#### 4.1.4. Melatonin

Melatonin (*N*-acetyl-5-methoxytryptamin), an amphiphatic neurohormone produced by the pineal gland, is able to cross all biological barriers, including the placenta and the blood–brain barrier [198,199]. Melatonin has potent antioxidant (ROS-scavenger activity) and anti-inflammatory properties. However, its production is significantly impaired in chronic renal failure [200]. This natural antioxidant functions either directly (independent of receptors to scavenge free radicals) or indirectly by regulating the expression and, therefore, activity of antioxidant enzymes including SOD, glutathione peroxidase, and glutathione reductase, as well as stimulates synthesis of glutathione [201,202]. When compared to Vitamins E and C, melatonin was found to be more effective in reducing the damage that occurs under high free radical conditions [203] (Table 2). The ability of melatonin to scavenge free hydroxyl radicals is attributed to a methoxy group (present at position 5 of the indole nucleus) and acetyl group of the side chain. Upon donation of electrons to scavenge –OH, melatonin is converted to indolyl cation radical, which is believed to neutralize superoxide radical. Its intracellular distribution and unique scavenging cascade brought about by its metabolites, contributes much to the high efficacy of melatonin. In addition, melatonin plays an important role in the control of reproductive functions, modification of immune system activity, and scavenging of radicals, thereby protecting the body against oxidative damage to DNA, proteins, and membranes [204]. Melatonin shows cytoprotective action against many highly toxic agents, including paraquat and carbon tetrachloride, which cause cell damage [205]. The anti-oxidative effects attributed to melatonin are clear because of its ability to protect haematopoietic cells from the damaging effects of lead, as well as its counteracting of the modification of expression of the hypothalamic gene (Per 1 and Per 2) by cadmium [206,207].

### 4.2. Essential Mineral Ions

#### 4.2.1. Selenium

Selenium (Se) is an essential trace element found in humans, animals, and some bacteria. In humans, its sources include meat, cereal grains, and fish. Average intake required for normal body functioning varies according to the age group; from 17 μg/day (children) to 45 μg/day (Adults) [208] (Table 3). As selenoproteins, it contributes significantly to the maintenance of essential biological functions. It exists in two forms: organic, as selenomethionine (SeMet), selenocysteine (SeCys) and metylselenocysteine (MeSeCys), and inorganic, as selenite and selenate [209]. It has been found to play an important role in at least 25 human selenoproteins by being part of the primary amino acid sequence as selenocysteine (SeCys) [210,211]. Among the series of selenoproteins, thioredoxin reductase and glutathione peroxidase representing selenoenzymes, play critical roles in the maintenance of cellular redox homeostasis [212]. By acting as an antimutagenic agent, it prevents malignant transformation of normal cells. As part of glutathione peroxidases (GSH-Pxs) and thioredoxin reductase, it is primarily associated with protecting DNA and other cellular components from oxidative damage [213]. It increases the antioxidant capacity of cells by enhancing the activity of superoxide dismutase associated with the scavenging of superoxide radicals, increasing glutathione reductase activity and, as such, glutathione content as part of its protection against heavy metals [214] (Table 2). Having the ability to enhance the levels of glutathione and Metallothioneins (MTs), its supplementation has been found to be associated with reversing the effect of different metals [215]. Its interaction with heavy metals such as mercury counteracts their negative effects via the formation of insoluble complexes that prevent them from exerting toxic effects on the body [216,217]. Along with, Se administration was found to have a positive effect in reducing the Pb and As toxicities through increased production of Selenoproteins, competition at key enzymes and through formation of inert Se–metal complexes [218]. In addition to its antioxidant property, it plays an important role in thyroid hormone metabolism and redox reactions [207,219]. Bronzetti *et al.* (2001) reported that, within certain limits, Se may have anticarcinogenic effects; however, at concentrations higher than those necessary for nutrition, it can have adverse effects by acting as a genotoxin and a carcinogen [220]. Besides being toxic in itself at higher concentrations, it has been found to enhance the toxicity imposed by Pb [218]. With greater chances to cause selenosis, higher intake to combat toxicity associated with metals such as mercury does not make it a preferable choice for therapy.

#### 4.2.2. *Iron*

Iron in the form of hemoproteins and iron-sulphur centres is the most abundant transition metal in the body. Having unusual flexibility to serve both as an electron donor and acceptor, it contributes significantly to cellular reactions within living organisms. However, the property that makes it an essential element also contributes to its potential toxicity by catalyzing reactions that generate free radicals. Required for various functions, average dietary intake ranges from 3–8 mg/day, except for pregnant women who usually require a higher amount for the proper development of the child [208] (Table 2). Biological reductants such as O_2_^−^ and ascorbic acid are known to regulate free iron (Fe^2+^) concentrations (0.2–0.5 lM) [170,221]. Representing an essential trace element, it is required for optimal physical and cognitive performance. Its essentiality to the human body stems from the fact that its deficiency continues to be the most prevalent micronutrient deficiency that acts as one of the leading factors behind disability and mortality worldwide [222]. In a survey conducted by the World Health Organization (WHO), 47.4% of children in the age group of 1–5 years, 25.4% of school children, 41.8% of pregnant and 30.2% of non-pregnant women, 12.7% of men, and 23.9% of the elderly population suffer from anaemia arising out of iron deficiency [223]. Upon stimulation by transition metal ions, Fe^2+^-mediated lipid peroxidation occurs after binding of Fe^2+^ to negatively charged phospholipids that results in alteration of the physical properties of the phospholipid bilayer and is followed by the initiation and propagation reactions of lipid peroxidation [224,225,226].

Fe^2+^ + ROOH → RO^•^ + OH^•^ + Fe^3+^

Fe^3+^ + ROOH → RO_2_^•^ + H^+^ + Fe^2+^

It is worth mentioning that iron as part of haemoglobin counteracts the toxic effects of Pb. Its deficiency has been found to be associated with exacerbation of the toxicity of Pb to haemopeotic system [227,228]. Iron deficiency of anaemic persons has been reported to demonstrate the significant impact of Pb compared with a normal iron load [227]. At the intestinal surface, it competes with the deleterious metals for uptake and, as such, prevents the body from toxic effects (Table 2). Its increased presence also helps in overcoming the competitive binding of heavy metals to active sites of enzymes that render them unable to perform their function [114,115]. A good example of this is of δ-aminolevulinic acid dehydratase (δ-ALAD) and ferrochelatase that are more prone to inhibition by Pb.

**Table 3:** Information regarding average dietary requirement and tolerable upper intake level of essential mineral ions in different age groups of humans.

Group	Age	Estimated Average Requirement				Tolerable Upper Intake Level			
		Selenium	Iron	Copper	Zinc	Selenium	Iron	Copper	Zinc
Children	1–3 yr	17 μg/d	3 mg/d	260 μg/d	2.5 mg/d	90 μg/d	40 mg/d	1000 μg/d	7 mg/d
	4–8 yr	23 μg/d	4.1 mg/d	340 μg/d	4 mg/d	150 μg/d	40 mg/d	3000 μg/d	12 mg/d
Males	9–18 yr	35–45 μg/d	5.9–7.7 mg/d	540–685 μg/d	7–8.5 mg/d	280–400 μg/d	40–45 mg/d	5000–8000 μg/d	23–34 mg/d
	19–70 yr	45 μg/d	6 mg/d	700 μg/d	9.4 mg/d	400 μg/d	45 mg/d	10000 μg/d	40 mg/d
Females	9–18 yr	35–45 μg/d	5.7–7.9 mg/d	540–685 μg/d	7–7.3 mg/d	280–400 μg/d	40–45 mg/d	5000–8000 μg/d	23–34 mg/d
	19–70 yr	45 μg/d	5–8.1 mg/d	700 μg/d	6.8 mg/d	400 μg/d	45 mg/d	10000 μg/d	40 mg/d
	Pregnancy (19–30 yr)	49 μg/d	22 mg/d	800 μg/d	9.5 mg/d	400 μg/d	45 mg/d	10000 μg/d	40 mg/d
	Lactation (19–30yr)	59 μg/d	6.5 mg/d	1000 μg/d	10.4 mg/d	400 μg/d	45 mg/d	10000 μg/d	40 mg/d

Source: National Academy of Sciences. Food and Nutrition Board, Institute of Medicine. National Academies Press, 2001. (For detailed reports: http://www.nap.edu./) [208]. Symbol representation: Yr: year; d: day.

#### 4.2.3. *Copper*

Copper, an important trace metal, acts as a cofactor for a variety of proteins and enzymes required for maturation of cytoplasmic cuproproteins and assembly of enzymes in different cell organelles (ceruloplasmin and tyrosinase in case of Golgi apparatus and cytochrome c oxidase with respect to mitochondria) (Table 2). Copper uptake occurs in a tightly regulated process through specific high-affinity plasma membrane copper transporters or low-affinity permeases [229,230]. Binding to chaperone proteins results in the transfer of copper to its final destination or any intermediate location from which its transport to other cell compartments or efflux out of cells can occur in cases in which concentration exceeds the optimum level. Acting as a cofactor for a wide range of metal-binding enzymes, it fluctuates between the oxidized Cu (II) and reduced Cu (I) forms. In humans, its average intake ranges between 260 and 700 μg/day [208] (Table 3). Although adequate intake of copper provides protection against lead, higher intake has been associated with increased lead absorption [231,232]. Its presence in excess amounts led to its involvement in the generation of highly reactive oxidative species (such as hydroxyl radicals), well known for their devastating effects in cells, particularly DNA damage and oxidation of proteins and lipids [233]. Cu(I) and Cu(II) that hold high affinity for protein sites having cysteine, methionine, and histidine side chains act as potential ligands that led to displacement of essential metal ions from their active sites, thereby resulting in the misfolding of proteins. As such, its uptake, followed by distribution and utilization, and finally excretion from the body needs to be tightly regulated [234,235].

#### 4.2.4. *Zinc*

Zinc, a ubiquitous trace element essential as a catalytic, structural, and regulatory ion, is indispensable for growth and development of microorganisms, plants, and animals [236]. Average human intake of zinc ranges between 2.5 and 10 mg/day [209] (Table 3). It is well known for its role as a cofactor for superoxide dismutase (SOD), and it protects biological structures from damage caused by free radicals by maintaining adequate levels of SOD and metallothioneins, as well as preventing interaction between chemical groups with iron (Table 2). It is a part of the zinc dependent thymic hormone that is essential for thymic functions such as T-cell maturation and differentiation [237]. Its antioxidant function is attributed to its function of blocking the negatively charged sites, thereby preventing lipid peroxidation. Its deficiency has mostly been associated with increase in the levels of lipid peroxidation of mitochondrial and microsomal membranes along with osmotic fragility of the erythrocyte membrane. Zinc-binding proteins such as metallothioneins (MTs) are present in virtually all living organisms. These proteins play an important role in zinc uptake, distribution, storage and release, and are protective *in situ* ations of stress (exposure to oxyradicals), exposure to toxic metals, and low Zn nutrition [238,239]. Zn as part of MTs improves excretion of metals such as Pb, As, *etc.* from the body (Table 2). In a study, Jamieson *et al.* (2006) found that marginal Zn deficiency enhances accumulation of Pb in bone, while its supplementation reduces its absorption and, as such, its accumulation in rats [240]. Co-administration of zinc and lead, which compete for similar binding sites on enzymes, results in the reverse inhibition of aminolevulinic acid dehydrogenase in male Wistar rats, suggesting that administration of zinc suppresses the toxic effects of lead [241,242]. In a similar study, supplementation of Zn was found to be associated with reduction in the effects of HgCl_2_ [243]. Possessing significant potential to displace Zn from Zn-metalloproteinases, it eliminates the effect of HgCl_2_ on neural development [244]. Zn along with Se has been associated with reduction in MeHg induced toxicity. All this indicates that it plays a protective role against the damage of different metals through reduction in absorption, competing for binding to enzymes and through induction of molecules such as MTs. Besides having positive effects, supplementation of Zn has also been found associated with displacement of essential metals to substitute normal physiological activities [228]. On one side, where the supplementation of Zn seems to provide protection against oxidative damage of iron in the instance of iron supplementation, long term or higher dosage treatment of Zn has been assocated wth the depletion of copper [217,245]. As such, a balanced approach in the supplementation of these metal ions is necessary to prevent unwanted complications.

### 4.3. Enzymatic Antioxidants

Chemically unstable compounds that carry free electrons (such as superoxide (O_2_^•**−**^) react with macromolecules, resulting in their destabilization by inducing damage to DNA, cellular proteins, and membrane lipids [246]. Superoxide dismutase effectively catalyzes the dismutation of superoxide to oxygen and hydrogen peroxide, which is, in turn, catabolised by catalase (located in the peroxisome) and peroxidase into oxygen and water. In humans, superoxide dismutase exists in three isoforms, cytosolic Cu, Zn-SOD, mitochondrial Mn-SOD, and extracellular SOD (EC-SOD) [247]. Cu, Zn-SOD is an approximately 32 kDa molecule that exists as a homodimer, with each component of the dimer having a dinuclear metal cluster composed of copper and zinc ions at the active site and activity independent of pH in the range of 5–9.5. Mitochondrial Mn-SOD, a homotetramer of ~96 kDa having one manganese atom per subunit, shows rapid transition from Mn(III) to Mn(II) and back to Mn(III) during the two step dismutation of superoxide [248]. Mitochondrial Mn-SOD is considered one of the most effective antioxidant enzymes that possess anti-tumour activity [249]. Similar to Mitochondrial Mn-SOD, extracellular superoxide dismutase (EC-SOD) having high affinity for glycosaminoglycans such as heparin and heparin sulphate is a secretory, tetrameric zinc and copper containing glycoprotein [248]. In mammals, its regulation occurs in a coordinated manner primarily by cytokines, rather than as a response to cellular oxidants. Antioxidant enzyme superoxide dismutase is present in nearly all cells exposed to oxygen, where it helps neutralize damaging reactions of superoxide, thereby protecting cells from the toxicity of superoxide (Table 2).

O_2_^•**−**^ → H_2_O_2_

Catalase possesses a very high turnover rate, with one molecule being able to convert ~6 million molecules of hydrogen peroxide to water and oxygen every minute.

2 H_2_O_2_ → O_2_ + H_2_O

Catalase generated hydrogen peroxide is acted upon by the enzyme glutathione peroxidase [Either selenium-independent (glutathione-S-transferase, GST) or by selenium-dependent (GPx) peroxidise]. All GPx enzymes are known to add two electrons to reduce peroxides by forming selenoles (Se–OH). GPx acts in conjunction with the tripeptide glutathione (GSH) as follows:

2 H_2_O_2_ + 2 GSH → 2 H_2_O + 2 O_2_ + GSSG → 2 GSH (Glutathione peroxidase)

Humans possess four different Se-dependent glutathione peroxidases [225]. Glutathione peroxidase 1 (GPx-1) is ubiquitously expressed as a major scavenger of H_2_O_2_ and lipid hydroperoxides. GPx-2 is epithelium specific and highly expressed in the GI tract. GPx-3, an extracellular glycosylated enzyme found in plasma, uses thioredoxin (TRx) and glutaredoxin (GRx) in addition to GSH as electron donors to reduce a broad range of hydroperoxides. GPx-4 present in cytosolic, mitochondrial and nuclear forms is associated with the prevention of membrane phospholipid oxidation.

### 4.4. Dietary Antioxidants

Cells possess a variety of antioxidant protection mechanisms against ROS, which are not present in excess. Accordingly, chelation therapy has been developed to reduce the toxic effects of heavy metals. Many thiol-containing molecules, including d-penicillamine, dimercaptosuccinic acid, 2,3-dimercaprol, and dimercaptopropane-sulfonic acid, have the ability for heavy metal complexation and are used as treatments for metal intoxications. However, owing to the potential health risks associated with these compounds, thiol-containing molecules from dietary sources have gained attention as potential detoxicants for heavy metals. In light of the oxidative damage caused by heavy metals, the following section highlights the importance of dietary substances that act along with cellular reductants to protect against oxidative damage and subsequent disease.

#### 4.4.1. α-Lipoic Acid

α-Lipoic acid (also known as thiotic acid, 1,2-dithiolane-3-pentanoic acid, 1,2-dithiolane-3-valeric acid, and 6,8-thiotic acid) is a naturally occurring compound synthesized by plants and animals, including humans. Its concentration in foods from animal sources is highest in metabolically active organs such as liver, kidney, and heart [250,251]. Though found in abundance in animal tissues, its concentration in plants varies; with spinach, broccoli, and tomatoes exhibiting the highest and garden pea and rice bran exhibiting the lowest [238]. Not acquired in a sufficient amount through diet, its synthesis follows the *de novo* path. Its synthesis occurs from octanoic acid and cysteine with the help of lipoic acid synthase [252]. α-Lipoic acid is unique among biological antioxidants in that it acts as an antioxidant in both oxidized and reduced forms [253]. Because of its amphipathic nature, its absorption from the small intestines is followed by rapid distribution to the liver through portal circulation and to other tissues of the body via systemic circulation [254]. In addition to performing antioxidant functions in the body, α-Lipoic acid acts as a non-vitamin nutritional coenzyme in the metabolism of proteins, carbohydrates, and fats. In the mitochondria, it functions as a co-factor to multienzyme dehydrogenase complexes such as pyruvate dehydrogenase (PDH) and α-ketoglutarate dehydrogenase, which are required for energy production by metabolizing carbohydrates, proteins, and fats [255] (Table 2). In the PDH complex, α-lipoic acid is covalently attached through an amide linkage to the ε-amino group of the lysine side chain of the dihydrolipoyl transacetylase component, thereby forming a lipoamide prosthetic group for acceptance and transfer of an acetyl group to CoA [256]. Owing to its localization in mitochondria, pyruvate dehydrogenase catalyzes the oxidative decarboxylation of pyruvate to acetyl CoA, a critical step in glucose metabolism via the citric acid cycle. A related metabolic function of α-lipoic acid is maintenance of blood glucose along with transport of blood glucose into the cell, which is explained in part by its function in glucose metabolizing enzymes [257].

Alpha-lipoic acid acts as a potential therapeutic agent for metal toxicity. Exhibiting rapid uptake in a variety of cells and tissues, supplementation of lipoic acid exogenously undergoes rapid reduction by NAD(P)H-dependent enzymes (thioredoxin reductase, cytosolic glutathione reductase, and mitochondrial dihydrolipoyl dehydrogenase ) to dihydrolipoate (DHLA), a reduced form of α-lipoic acid which possesses superior antioxidant potential compared to α-lipoic acid due to there being two free thiol (–SH) groups [258]. As both α-lipoic acid and DHLA possess chelating properties for metal ions, they are beneficial against iron, cadmium, copper, and mercury poisoning [9]. α-lipoic acid and its metabolite DHLA can scavenge reactive oxygen species (ROS) and reactive nitrogen species (RNS) such as hydroxyl radicals, singlet oxygen species, H_2_O_2_, peroxyl radicals, superoxide radicals, and peroxynitrite radicals [259] (Table 2). Compared to the oral lethal dose (LD_50_) of 1130 and 502 mg/kg and IP LD_50_ of 200 and 160 mg/kg for rat and mice, the tolerance level of humans for α-lipoic acid was several grams following oral administration [259]. Indeed, no serious side effects have been reported even after administration of approximately 1 g of lipoic acid. Inside the body, α-lipoic acid was found to be more effective in chelating lead while its metabolite DHLA was able to chelate both lead and mercury [242]. The No Observed Adverse Effect Level (NOAEL) for α-lipoic acid was 60 mg/kg·bw/day [260]. Mean plasma half-life of α-lipoic acid is approximately 30 min following both intravenous (iv) or oral administration [261]. Ali *et al.* (2015) reported that administration of α-lipoic acid leads to a decrease in the level of lipid peroxidation in plasma, liver, and brain [262]. This property may be attributed to its bioavailability, which it employs in counteracting oxidative species directly. Unlike 2,3-dimercaptosuccinic acid (DMSA) and 2,3-dimercapto-1-propanesulfonic acid (DMPS), α-lipoic acid can cross the blood–brain barrier. DHLA is also associated with regeneration of major physiological antioxidants, possessing the ability to reduce oxidised forms of ascorbic acid (Vitamin C), glutathione, and α-tocopherol (Vitamin E) [263,264]. In short, α-lipoic acid acts by multiple mechanisms both pharmacologically and physiologically. Pharmacologically, α-lipoic acid is used in the treatment of diseases such as diabetic neuropathy, and physiologically, it is used as an antioxidant. In addition, it directly terminates free radicals, chelates and metal ions, and regenerates endogenous antioxidants such as glutathione along with Vitamin C and Vitamin E.

#### 4.4.2. Vitamin C

Citrus fruits and green leafy vegetables in food from plants and kidney along with liver from an animal source serve as the main sources of Vitamin C. Vitamin C (ascorbic acid), a di-acid (AscH_2_) with two ionisable hydroxyl groups, is a very important and powerful antioxidant that works in aqueous environments inside the body. Transport of dehydroascorbic acid (Asc), an oxidized form of Vitamin C, occurs by means of glucose transporters, while that of its reduced form is mediated by sodium-dependent amino acid transporters [265,266]. Having the limitation of becoming saturated, the extent of its absorption from the GI tract ranges between 80% and 90% at an intake of 30–180 mg/person/day. During pregnancy, oral intake of up to 1 g/kg·bw/day had no detrimental effect in rats and mice [267]. Similarly, in addition to normal dietary intake, supplementary doses of approximately 1 g/person per day had no adverse effect on the GI tract in humans. However, with an increase in the dose (3–4 g/person per day or greater), extent of GI problems subsequently increase [268]. Compared to ascorbic acid, absorption of ascorbyl palmitate occurs by passive diffusion. Being lipophilic, it enters the bloodstream unchanged and also penetrates cell membranes including the blood–brain barrier [269]. Under normal physiological conditions, Vitamin C exists primarily as AscH^−^ (99.9%), with only trace amounts present as AscH_2_ (0.05%) and Asc^2−^ (0.004%). Upon reaction with radicals, AscH^−^ acts as a donor antioxidant to produce the resonance stabilised tricarbonyl ascorbate free radical (AscH^•^). AscH^•^ is not protonated because its pK value is –0.86, hence it occurs in the form of semi-dehydroascorbate radical (Asc^•−^).

AscH_2_ ←→ AscH^−^ → AscH^•^

AscH^•^ ←→ Asc^•−^

By estimating the level of semi-dehydroascorbate radical (Asc^•−^), we can obtain a good measure of the degree of oxidative stress in biological systems [270,271]. Being relatively un-reactive, semi-dehydroascorbate radical (Asc^•−^) through reaction with other free radicals leads to chain termination of free radical reaction (Table 2).

2 Asc^•−^ + H^+^ → AscH^−^ + Asc^•^

ROO^•^ + AscH^−^ → ROOH + Asc^•^

Asc^•^ + O_2_ → Asc + O_2_^•−^

O_2_^•−^ + ROO^•^ + H^+^ → O_2_ + ROOH (Chain termination)

Asc^•−^ + ROO^•^ + H^+^ → Asc + ROOH (Chain termination)

Ascorbic acid and GSH, which are among the most active reducing substances in living tissues, show strong interrelationships during redox cycling under *in vivo* conditions. Toxicity arising as a result of GSH deficiency was delayed on administration of ascorbate, while on return it was found to facilitate two-electron reduction that led to regeneration of ascorbic acid from its oxidized form, dehydroascorbate; with GSH-ascorbic acid conjugate as the intermediate product of the reaction [272,273,274]. Throughout the process of conversion, one GSH molecule acts as a nucleophile that attacks the central carbonyl of dehydroascorbate, and involvement of another leads to the production of ascorbate and GSSG.

As an active reducing agent, ascorbic acid plays an important role in the metabolism and detoxification of many endogenous and exogenous compounds [275]. In addition to scavenging singlet oxygen, superoxide, hydroxyl, peroxyl radical and hypochlorous acid, it acts as an excellent donor of electrons to free radicals such as hydroxyl and super oxide radicals, and thereby quenches their activity. It efficiently inhibits lipid peroxidation by scavenging reactive oxygen species (ROS) via rapid electron transfer [276,277,278]. In the majority of *in vivo* studies, supplementation of Vitamin C has shown pronounced beneficial effects as indicated by a reduction in markers of oxidative DNA, lipid and protein damage. However, *in vitro* studies have revealed that Vitamin C either exhibits no effect or shows inhibition of lipid peroxidation mediated by transition metal (Fe, Cu) ions. An observed reduction in the incidence of stomach cancer by Vitamin C supplementation was likely due to its inhibitory action against the generation of *N*-nitroso compounds via interruption of the reaction between nitrites and amine groups [279]. Vitamin C has also been found to exhibit cooperative behaviour with Vitamin E in the regeneration of α-tocopherol from α-tocopherol radicals in membranes and lipoproteins [280,281]. In addition, co-joint supplementation of ascorbic acid and α-tocopherol not only alters the TNF-α level, but inhibits activation of the caspase cascade and, therefore, the extent of damage caused to DNA in animals exposed to arsenic [282]. Along with this, co-administration of Vitamin C and thiamine has been found to enhance the efficacy with which chelating agents facilitate metal excretion, particularly that of lead, thereby supporting the view that ascorbic acid acts as a detoxifying agent by forming a poorly ionised but soluble complex with lead [283,284].

#### 4.4.3. Carotenoids

Similar to other common antioxidants, carotenoids are pigments commonly found in plants and microorganisms. Humans are unable to synthesize them, and therefore acquire them mainly through diet [285]. Based on structure, they are classified according to two groups: carotenes, including β-carotene and lycopene, and xanthophylls, including astaxanthin, lutein, zeaxanthin, canthaxanthin, capsorubin, and violaxanthin [286,287]. Astaxanthin, having more importance with respect to humans, is found predominantly in a marine environment (microalgae, plankton, *etc.*). It has both hydrophilic and lipophilic properties. Because it cannot be synthesized and there is lesser chance of conversion to Vitamin A, it is safer as its accumulation does not lead to hyper-vitamin toxicity [288]. Its approval for use as a dietary supplement (nutraceutical) has led to its ambiguous use in therapeutic purposes [289,290]. Although its bioavailability in humans was observed at a dose of 100 mg, it shows increased absorption when taken as lipid based formulations [290]. Having conjugated double bonds causes it to act as a strong antioxidant. By donating electrons to free radicals, it can terminate a free radical chain reaction by quenching various ROS and RNS in a wide variety of living organisms [291] (Table 2). Compared to lutein, lycopene, and β-carotene, it exhibits higher antioxidant activity [292,293]. Antioxidant activity of astaxanthin was 10 times more than that of zeaxanthin, lutein, canthaxanthin, and β-carotene, and 100 times higher than that of α-tocopherol [294]. Its presence has also been associated with increase in antioxidant enzymes such as catalase, superoxide dismutase, and peroxidase [295].

β-carotene, a precursor of retinol (Vitamin A), made of isoprene units that impart it with a lipid soluble nature, shares properties somewhat analogous to those of α-tocopherol (Vitamin E). Following oxidative stress, suppression of the activation and production of NF-kB, interleukin-6 (IL-6), and TNF-α inflammatory cytokines by β-carotene treatment suggests a protective effect corresponding to β-carotene in cells [296]. The ability of the conjugated double-bonded structure to delocalise unpaired electrons [297] contributes to physical quenching of the singlet oxygen and to the chemical reactivity of β-carotene that led to neutralization of free radicals such as the peroxyl (ROO^•^), hydroxyl (^•^OH), and superoxide (O_2_^•−^). Significant protection of lipids from peroxidative damage occurs at high concentrations of carotenoids. Previous studies have shown that scavenging of lipid ROO^•^ by β-carotenes proceeds through adduct formation and/or hydrogen abstraction rather than via processes involving electron-transfer mechanisms [298]. Following cadmium chloride intoxication in animals, β-carotene was found to be beneficial in recovering the activity of glutathione-S-transferase. In addition, other studies have provided clear evidence indicating that β-carotenoid supplementation leads to prevention or inhibition of certain types of cancer, arthrosclerosis, age-related muscular degeneration, and other diseases.

## 5. Conclusions

Agents responsible for multiple human complications vary grossly in their physiochemical properties, and metals are no exception. After entering an ecosystem, metals induce a broad range of physiological, biochemical, and behavioural dysfunctions via induction of oxidative stress in humans. Oxidative and nitrative stress developed in response to toxicants plays an important role in damaging biomolecules, as well as disrupting signalling pathways, which in turn leads to pathogenesis of multiple human diseases. Despite the protection afforded by the cellular redox environment in biological systems, its disruption due to exogenous stimuli or endogenous metabolic alteration leads to increased intracellular ROS/RNS levels. Buffering and muffling reactions between ROS/RNS generation and elimination to redress the deleterious effects caused by oxidative stress are maintained by complex antioxidant (enzymatic and non-enzymatic) systems. In terms of a reactivity standpoint, the enzymatic antioxidant system constitutes the first line of defence, followed by reduced thiols and low molecular weight antioxidants and then by a broad range of products from dietary sources. Defense systems for overcoming the deleterious effects of oxidative and nitrative stress generated by production of reactive oxygen species (ROS) and reactive nitrogen species (RNS) are essential to maintenance of cellular homeostasis. However, depletion of the cellular antioxidant pool characterized by (a) increased ROS and RNS production; (b) depletion of free-radical scavengers (Vitamins E and C) and cellular antioxidants (largely GSH); and (c) inhibition of the activity of enzymes such as (GPx) glutathione peroxidase, GSH-reductase, GSH-transferase, catalase (CAT) and superoxide dismutase (SOD) that contribute significantly to the metabolism and detoxification of reactive oxygen species (ROS). Having grave consequences within the bodies of living organisms, maintaining the availability of essential and controlled distribution of toxic metal ions is an efficient means of protection against the deleterious effects of heavy metals. Accordingly, an improved understanding of the counter-productive and beneficial defensive mechanisms of dietary antioxidants support their role in therapeutics for metal induced oxidative stress. To bridge this knowledge gap, studies pertaining to elucidation of molecular mechanisms involved in imparting toxicity imposed by heavy metals are essential. In addition, detailed mechanistic studies underlying the beneficial effects of dietary antioxidants for their optimum dosage and duration of treatment will be helpful in the development of combinatorial strategies as part of effective treatment regimes for better clinical recovery in metal intoxication cases.
